# Hybrid assembly with long and short reads improves discovery of gene family expansions

**DOI:** 10.1186/s12864-017-3927-8

**Published:** 2017-07-19

**Authors:** Jason R. Miller, Peng Zhou, Joann Mudge, James Gurtowski, Hayan Lee, Thiruvarangan Ramaraj, Brian P. Walenz, Junqi Liu, Robert M. Stupar, Roxanne Denny, Li Song, Namrata Singh, Lyza G. Maron, Susan R. McCouch, W. Richard McCombie, Michael C. Schatz, Peter Tiffin, Nevin D. Young, Kevin A. T. Silverstein

**Affiliations:** 1 J. Craig Venter Institute, 9714 Medical Center Drive, Rockville, MD 20850 USA; 2 Department of Plant Biology, University of Minnesota, Saint Paul, MN USA; 3 National Center for Genome Resources, Santa Fe, NM USA; 4 Cold Spring Harbor Laboratory, Harbor, Cold Spring, NY USA; 5 Stanford School of Medicine, Stanford, CA USA; 6 National Human Genome Research Institute, Bethesda, MD USA; 7 Department of Agronomy and Plant Genetics, University of Minnesota, St. Paul, MN USA; 8 Department of Plant Pathology, University of Minnesota, St. Paul, MN USA; 9 Department of Computer Science, Johns Hopkins University, Baltimore, MD USA; 10 School of Integrative Plant Sciences, Plant Breeding and Genetics section, Cornell University, Ithaca, NY 14850 USA; 11 Departments of Computer Science and Biology, Johns Hopkins University, Baltimore, MD USA; 12 Minnesota Supercomputing Institute, University of Minnesota, Minneapolis, MN USA

**Keywords:** Genome assembly, Hybrid assembly pipeline, Tandem repeats, *Medicago truncatula*

## Abstract

**Background:**

Long-read and short-read sequencing technologies offer competing advantages for eukaryotic genome sequencing projects. Combinations of both may be appropriate for surveys of within-species genomic variation.

**Methods:**

We developed a hybrid assembly pipeline called “Alpaca” that can operate on 20X long-read coverage plus about 50X short-insert and 50X long-insert short-read coverage. To preclude collapse of tandem repeats, Alpaca relies on base-call-corrected long reads for contig formation.

**Results:**

Compared to two other assembly protocols, Alpaca demonstrated the most reference agreement and repeat capture on the rice genome. On three accessions of the model legume *Medicago truncatula*, Alpaca generated the most agreement to a conspecific reference and predicted tandemly repeated genes absent from the other assemblies.

**Conclusion:**

Our results suggest Alpaca is a useful tool for investigating structural and copy number variation within de novo assemblies of sampled populations.

**Electronic supplementary material:**

The online version of this article (doi:10.1186/s12864-017-3927-8) contains supplementary material, which is available to authorized users.

## Background

Tandemly duplicated genes are important contributors to genomic and phenotypic variation both among and within species [1]. Clusters of tandemly duplicated genes have been associated with disease resistance [2], stress response [3], and other biological functions [4, 5]. Confounding the analysis of tandem repeats in most organisms is their underrepresentation in genome assemblies constructed from short-read sequence data, typically Illumina reads, for which the sequence reads are shorter than repeats [6–9].

The ALLPATHS-LG software [10] overcomes some of the assembly limitations of short-read sequencing by clever combination of Illumina paired end reads from both short-insert and long-insert libraries. Applied to human and mouse genomes, the ALLPATHS assembler produced assemblies with more contiguity, as indicated by contig N50 and scaffold N50, than had been attainable from other short-read sequence assemblers. ALLPATHS also performs well on many other species [11, 12]. The ALLPATHS assemblies approached the quality of Sanger-era assemblies by measures such as exon coverage and total genome coverage. However, the ALLPATHS assemblies captured only 40% of genomic segmental duplications present in the human and mouse reference assemblies [10]. Similarly, an ALLPATHS assembly of the rice (*Oryza sativa* Nipponbare) genome [13] was missing nearly 12 Mbp of the Sanger-era reference genome, including more than 300 Kbp of annotated coding sequence. These findings illustrate the potential for loss of repeat coding sequence in even the highest quality draft assemblies constructed exclusively from short-read sequence data.

Long-read sequencing offers great potential to improve genome assemblies. Read lengths from PacBio platforms (Pacific Biosciences, Menlo Park CA) vary but reach into the tens of kilobases [9]. The base call accuracy of individual reads is about 87% [14] and chimera, i.e. falsely joined sequences, can occur within reads [15]. Although low base call accuracy and chimeric reads create challenges for genome assembly, these challenges can be addressed by a hierarchical approach [9] in which the reads are corrected and then assembled. The pre-assembly correction step modifies individual read sequences based on their alignments to other reads from any platform. The post-correction assembly step can use a long-read assembler such as Celera Assembler [16–18], Canu [19], HGAP [20], PBcR [21], MHAP [22], or Falcon [23]. Because most of the errors in PacBio sequencing are random, PacBio reads can be corrected by alignment to other PacBio reads, given sufficient coverage redundancy [24]. For example, phased diploid assemblies of two plant and one fungal genome were generated by hierarchical approaches using 100X to 140X PacBio [25] and a human genome was assembled from 46X PacBio plus physical map data [23]. Despite the potential of long-read assembly, high coverage requirements increase cost and thereby limit applicability.

Several hybrid approaches use low-coverage PacBio to fill gaps in an assembly of other data. The ALLPATHS pipeline for bacterial genomes maps uncorrected long reads to the graph of an assembly in progress [26]. SSPACE-LongRead, also for bacterial genomes, maps long reads to contigs assembled from short reads [27]. PBJelly [28] maps uncorrected long reads to the sequence of previously assembled scaffolds and performs local assembly to fill the gaps. In tests on previously-existing assemblies of eukaryotic genomes, PBJelly was able to fill most of the intra-scaffold gaps between contigs using 7X to 24X long-read coverage [28]. These gap filling approaches add sequence between contigs but still rely on the contig sequences of the initial assemblies. As such, gap filling may not correct assembly errors such as missing segmental duplications or collapsed representations of tandemly duplicated sequence. Long reads that span both copies of a genomic duplication, including the unique sequences at the repeat boundaries, are needed during the initial contig assembly to avoid the production of collapsed repeats.

We developed a novel hybrid pipeline named Alpaca (ALLPATHS and Celera Assembler) that exploits existing tools to assemble Illumina short-insert paired-end short reads (SIPE), Illumina long-insert paired-end short reads (LIPE), and PacBio unpaired long reads. Unlike other approaches that use Illumina or PacBio sequencing for only certain limited phases of the assembly, Alpaca uses the full capabilities of the data throughout the entire assembly process: 1) contig structure is primarily formed by long reads that are error corrected by short reads, 2) consensus accuracy is maximized by the highly accurate base calls in Illumina SIPE reads, and 3) scaffold structure is enhanced by Illumina LIPE that can provide high-coverage connectivity at scales similar to the PacBio long reads. We targeted low-coverage, long-read data in order to make the pipeline a practical tool for non-model systems and for surveys of intraspecific structural variation.

We evaluated the performance of Alpaca using data from *Oryza sativa* Nipponbare (rice), assembling the genome sequence of the same *O. sativa* Nipponbare accession used to construct the 382 Mbp reference, which had been constructed using clone-by-clone assembly, Sanger-sequenced BAC ends, physical and genetic map integration, and prior draft assemblies [29]. We also sequenced and assembled three accessions of *Medicago truncatula*, a model legume, and compared these to the *M. truncatula* Mt4.0 reference assembly of the A17 accession [30]. The Mt4.0 reference had been constructed using Illumina sequencing, an ALLPATHS assembly, Sanger-sequenced BAC ends, a high-density linkage map, plus integration of prior drafts that integrated Sanger-based BAC sequencing and optical map technology [31].

For the *Medicago* analyses where no high quality reference sequence was available for the accessions whose genomes we assembled, we focused our evaluation on the performance of Alpaca on large multigene families that play important roles in plant defense (the NBS-LRR family) and in various regulatory processes involving cell to cell communications (the Cysteine-Rich Peptide, or CRP, gene family). Members of these multigene families are highly clustered; the reference genome of *M. truncatula* harbors more than 846 NBS-LRR genes, with approximately 62% of them in tandemly arrayed clusters and 1415 annotated Cysteine-Rich Peptide (CRP) genes, with approximately 47% of them in in tandemly arrayed clusters. Resolving variation in gene clusters like these is crucial for identifying the contribution of copy number variation (CNV) to phenotypic variation as well as understanding the evolution of complex gene families.

## Results

### Rice genome assembly

The rice Nipponbare genome, which offers an independent reference, was used to evaluate assembly methods. An ALLPATHS assembly was generated from Illumina short reads and these data were used with 33X PacBio long reads to generate PBJelly and Alpaca assemblies. The Alpaca process included correcting long reads by (1) using Celera Assembler [16–18] to generate unitigs (preliminary contigs) from Illumina short-insert paired ends, (2) mapping unitigs to raw long reads with Nucmer [32], and (3) correcting the long read base calls with ECTools [33]. For separate evaluation of the correction step, the raw and corrected long reads were aligned to the reference. On average, raw reads aligned at 82% identity over 89% of their length, while corrected reads aligned at 98% identity over 95% of their length. The assemblies were evaluated several ways starting with size. Compared to ALLPATHS, the hybrid methods increased the total span and NG50 of contigs and scaffolds. Contig NG50 was 21 Kbp for ALLPATHS but 69 Kbp and 67 Kbp for PBJelly and Alpaca respectively. Scaffold NG50 was 192 Kbp for ALLPATHS but 223 Kbp and 255 Kbp for PBJelly and Alpaca (Additional file 1).

Assembled scaffolds were aligned to the reference with Nucmer [32]. Compared by alignment length N50, the PBJelly and Alpaca alignments were nearly twice as large as those of ALLPATHS; Table 1. Alpaca alignments had the largest sum of bases, average size, and maximum size, and these results held whether alignments were filtered for best alignment per assembly position, per reference position, or both (Additional file 2). In alignments filtered for best alignment per assembly position, each assembly had an alignment span that exceeded its contig span. The excess indicates sequence present in the reference at higher copy than in the assembly, i.e. collapsed repeats. The hybrid methods reduced this excess: 46 Kbp for ALLPATHS, 37 Kbp for PBJelly, and 35 Kbp for Alpaca. These alignments were further filtered for minimum 99% average identity to reduce repeat-induced mis-alignments. With these alignments, the Alpaca assembly provided the most reference coverage: 82% by ALLPATHS, 79% by PBJelly, and 88% by Alpaca. Thus, the Alpaca consensus provides the most 99%-identity reference coverage of the three assemblies tested. Note the PBJelly assembly could be expected to include low-quality consensus in regions corresponding to the ALLPATHS gaps because PBJelly was given low-coverage uncorrected PacBio reads and not supplemented with consensus polishing, e.g. [34].

**Table 1:** Change in reference agreement attributable to hybrid assembly methods

Source	Metric	ALLPATHS	PBJelly	Alpaca
	Agreement			
Nucmer	Alignment N50	20,539	+86%	+99%
ATAC	Alignment N50	174,306	+12%	+27%
Quast	NGA50	86,432	0%	+30%
	Disagreement			
Quast	Misassemblies	3784	+50%	−17%
Quast	Local misassemblies	9444	−21%	−43%
Quast	Misassembled contigs	1423	+17%	−13%

The rice Nipponbare genome was assembled with ALLPATHS and then re-assembled with the PBJelly and Alpaca hybrid methods. All assemblies were compared to the independently derived reference and reference agreement was measured relative to the ALLPATHS level. **Top**: the sizes of alignments to the reference characterized by N50. Nucmer alignments are bounded by contigs while ATAC “M c” alignments can span intra-scaffold gaps. Quast NGA adjusted N50 after breaking at mis-assemblies. **Bottom**: Quast uses Nucmer alignments to infer global and local mis-assemblies, where the former involve spans or transpositions of 1Kbp or larger

The assemblies were further analyzed with the ATAC glocal aligner [17] and Quast [35] which uses Nucmer. Table 1 presents the ALLPATHS-reference agreement as a baseline with the PBJelly and Alpaca gains and losses shown relative to ALLPATHS. Both hybrid methods increased the sizes of reference alignments but Alpaca demonstrated larger gains under each alignment test. Of mis-assemblies inferred from alignments using Quast, Alpaca reduced all three mis-assembly metrics. PBJelly saw less reduction of local mis-assembly and it actually increased the numbers of large mis-assemblies and mis-assembled contigs relative to ALLPATHS. Additional files provide Nucmer (Additional file 2), ATAC (Additional file 3), and Quast (Additional file 4) results.

To evaluate the efficacy of Alpaca at identifying tandemly duplicated genes, we first identified repeats through “alignment-to-self” analysis, filtering for minimum 95% identity and maximum 1 Mbp separation, in Nucmer alignments parameterized for repeat detection. The process identified 65,874 repeat sequence pairs in the rice reference. The process was repeated on the ALLPATHS, PBJelly, and Alpaca scaffolds. None of these assemblies captured as many repeats, in part because the assembled scaffolds are much smaller than the reference chromosomes. Alpaca contained the most repeats (9916) and its repeat collection had the largest N50 (1397 bp) which was twice as large as the PBJelly N50 (Additional file 5).

To evaluate recall, scaffolds from the ALLPATHS, PBJelly, and Alpaca assemblies were aligned to the reference and evaluated for their coverage of repeats in the reference. A pair of reference repeats was classified as “one scaffold” if both reference repeat units were over 50% covered by one scaffold, or as “two scaffolds” if each unit was over 50% covered by a different scaffold, or as “underrepresented” if either repeat unit was not 50% covered. Note the underrepresented category can include repeats that were partially assembled. For example, the largest rice repeat, consisting of two identical 70 Kbp units, was classified as underrepresented in all three assemblies. Though each assembly did have several alignments to the repeat, no single alignment surpassed the 35 Kbp threshold. Note also that the “one scaffold” category allows each assembly to cover multiple reference repeats with a single collapsed repeat in the assembly. Thus, in the results below, each assembly put more reference tandem repeats in the “one scaffold” category than were detected by the “alignment-to-self” analysis of that assembly.

Using a 2Kbp threshold to distinguish long and short repeats, the reference had 4734 pairs of long repeats and 61,140 pairs of short repeats. The ALLPATHS assembly captured a majority of the short repeats, leaving only 16% underrepresented, while leaving 93% of long repeats underrepresented (Table 2A). Compared to ALLPATHS, both hybrid assemblies captured higher portions of the long and short repeats, leaving fewer underrepresented. On short repeats, the PBJelly assembly captured more pairs than Alpaca, though much of the gain was in repeat pairs captured by two scaffolds. Of long repeats, the Alpaca assembly captured 52% in one scaffold and 88% in one or two scaffolds and these rates were much higher than in the other assemblies.

**Table 2:** Analysis of short and long tandem repeats in three assemblies of rice

A	Category	ALLPATHS	PBJelly	Alpaca
Unit > =2Kbp	One scaffold	2.4%	6.9%	51.6%
	Two scaffolds	4.2%	25.3%	36.5%
	Underrepresented	93.4%	67.8%	11.8%
	Total	4734	4734	4734
Unit < 2Kbp	One scaffold	71.3%	81.8%	80.1%
	Two scaffolds	12.8%	12.0%	6.7%
	Underrepresented	15.9%	6.2%	13.2%
	Total	61,140	61,140	61,140
B	Category	ALLPATHS	PBJelly	Alpaca
Unit > =2Kbp	One chromosome	43.9%	32.1%	61.3%
	Two chromosomes	0.9%	1.1%	4.7%
	Underrepresented	55.3%	66.8%	33.9%
	Total	114	184	548
Unit < 2Kbp	One chromosome	61.6%	58.1%	73.3%
	Two chromosomes	4.1%	4.2%	1.9%
	Underrepresented	34.3%	37.7%	24.7%
	Total	8079	8034	9368

A. Repeat pairs on reference chromosomes were classified by whether both repeated units were 50% covered by alignments to one scaffold, two scaffolds, or were “underrepresented”, in each of three assemblies. B. Conversely, repeat pairs on assembled scaffolds were classified by whether they were 50% covered by alignments to chromosomes in the reference. There are fewer total repeats in (B) because the number of same-scaffold repeats is lower in each assembly than the number of same-chromosome repeats in the reference

Of tandem repeats captured by one scaffold, some were captured within a single alignment, indicating agreement of repeat positioning and any intervening sequence. For repeats of any size captured by a single alignment to the reference, ALLPATHS captured 2425, PBJelly captured 4788, and Alpaca captured 6413.

To assess the reliability of repeats present in each assembly, the preceding analysis was repeated while reversing the roles of reference and assembly. In other words, the process gathered same-scaffold repeat pairs at 95% identity, and classified pairs according to alignments to reference chromosomes covering 50% of each repeat unit. The Alpaca assembly contained the most long and short repeat pairs (Table 2B). Of same-scaffold repeat pairs that aligned to the same chromosome, the Alpaca assembly had the highest portion for both long and short repeats. However, Alpaca had 26 long pairs (5%) that aligned to different chromosomes, indicating some false duplicates within its scaffolds.

### Medicago genome assembly

To explore the utility of Alpaca for investigating intraspecific variation in tandem repeats, the genomes of three wild accessions of *M. truncatula* were each sequenced and assembled by the three assembly processes. Compared to the 413 Mbp chromosome sequence span of the Mt4.0 reference assembly, all three ALLPATHS assemblies had slightly smaller span (Additional file 6). The six hybrid assemblies had approximately 10% larger scaffold spans than the corresponding short-read assembly. The hybrid assemblies had 3-fold to 6-fold larger contig NG50 than the short-read assembly. The PBJelly contig N50 was larger than the Alpaca on two of the three genomes. The PBJelly scaffold N50 was slightly larger than the ALLPATHS on all three genomes while the Alpaca scaffold N50 was smaller. This result is consistent with the observations that PBJelly builds on ALLPATHS scaffolds while Alpaca does not necessary recapitulate them.

The lack of a sufficiently close reference precluded most alignment-based confirmation of the various scaffold conformations. However, using the reference Mt4.0 assembly from the A17 accession, Nucmer put between 219 and 347 Mbp of the reference in local alignments with our assemblies. While each assembled accession is expected to have sequence and structural differences with the reference accession, local alignments between accessions should reflect the amount of sequence that is both shared across accessions and correctly assembled. All three assembly strategies generated their largest alignments on accession HM056 and their smallest alignments for HM340, consistent with earlier SNP analysis indicating HM056 is most closely related to A17 and HM340 is most diverged from A17 [36]. On the three accessions of *Medicago*, the hybrid assemblies’ alignment N50 was 12% to 25% larger than that of ALLPATHS and Alpaca had the largest sum of bases aligned at thresholds of 90 through 99% identity (Additional file 7).

To assess the tandem repeat content, tandem repeats were counted by the “alignment-to-self” method described above. Consistent with the results on rice, the Alpaca assemblies contained more repeats than the other assemblies and more large repeats in particular (Table 3, Additional file 8). The *Medicago* repeat content, however, could not be directly validated due to the lack of same-accession reference sequences. After mapping uncorrected long reads to the Alpaca assemblies of the same accessions, we found some repeats with low coverage indicative of overrepresentation in the assembly. However, coverage distributions across various sizes of repeats did not reveal systematic problems (Additional file 9).

**Table 3:** Counts and lengths of alignments to the reference

	Accession	ALLPATHS	PBJelly	Alpaca
Count, 2Kbp or longer	HM034	296	553	2058
	HM056	257	436	1652
	HM340	273	443	1947
Count, Under 2Kbp	HM034	14,990	14,911	18,888
	HM056	14,665	14,110	14,603
	HM340	18,206	17,225	19,334
Average length	HM034	294	388	769
	HM056	291	373	767
	HM340	271	336	730

In each of three *Medicago* accessions assembled three ways, the Alpaca assembly contained the most repeats and the largest average repeat length

### Medicago gene clusters

**Fig. 1:**
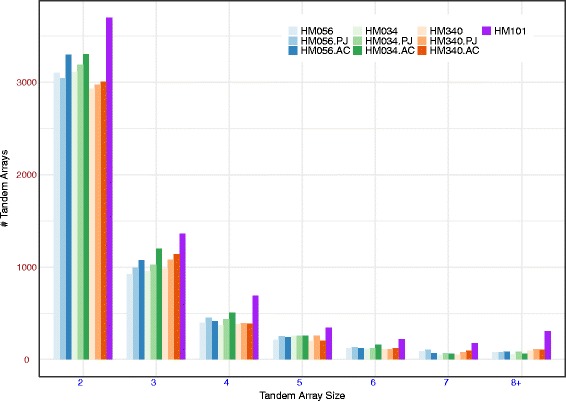
Tandemly array counts per assembly. Teh assemblies of four *Medicago truncatula* accessions were analyzed for gene cluster content. Each vertical bar of the histogram indicates the number of tandem gene clusters. Left to right per cluster: light blue = HM056 ALLPATHS, blue = HM056 PBJelly, dark blue = HM056 Alpaca, light green = HM034 ALLPATHS, green = HM034 PBJelly, dark green = HM034 Alpaca, light orange = HM340 ALLPATHS, orange = HM340 PBJelly, dark orange = HM340 Alpaca, and purple = the Mt4.0 reference assembly of the A17 (HM101) accession

The *Medicago* Alpaca scaffolds were assessed for gene cluster content and compared to the ALLPATHS scaffolds, PBJelly scaffolds, and the Mt4.0 reference assembly. To assess the gene cluster content, the *Medicago* assemblies were searched in protein space in order to find diverged gene copies. As expected, this search revealed more repeats per assembly than the stringent search used in Table 3. Counts of genes that occur in clusters of two or more genes are shown in Fig. 1 for each of ten assemblies. At every cluster size shown, the reference genome contains more clusters than all of our assemblies of the other accessions. This is most likely due to greater connectedness in the chromosome-length pseudomolecule sequences of the Mt4.0 reference and indicates that the assemblies are missing some tandem arrays. For most array sizes and accessions, the Alpaca assembly contained as many or more clustered genes as the ALLPATHS and PBJelly assemblies although the differences were small. A qualitatively similar pattern was observed for each of several gene families (Additional file 10).

Three large gene families with members often found in tandem arrays all have more identified members in the Alpaca than ALLPATHS assemblies: CRPs, TEs, and NBS-LRRs (Additional file 11, Additional file 12 and Additional file 13). The greatest difference between Alpaca and ALLPATHS identifications in gene clusters was seen for TEs where Alpaca identified 30–50% more TEs than ALLPATHS. Interestingly, the difference is attributable to specific TE subclasses. For instance, repeat family signatures RVT_1, rve and Retrotrans_gag have 60–100% more members, whereas other repeat families show little quantitative difference between assemblies. For CRPs, Alpaca identified only 1–5% more genes than ALLPATHS. However, for three CRP subgroups (CRP0355, CRP3710 and CRP4180), the Alpaca assemblies show an average membership increase of 30%, 340% and 190%, respectively, compared to the ALLPATHS. Phylogenetic trees for these CRP groups show recent (highly similar) accession-specific clade expansions, each captured in one or a few Alpaca scaffolds (Additional file 14, Additional file 15 and Additional file 16).

The largest expanded CRP cluster from the subgroup CRP3710 was examined in more detail. The Mt. 4.0 reference genome contains a single annotated CRP3710 gene, Medtr8g031540, with an identical unannotated pseudogene (missing the first 6 bp of the CDS) 19,678 bp away. No other genes with remote similarity exist in the genome. The HM034 Alpaca assembly has 29 tandem copies of this gene (>97% identity) on two scaffolds including two partial (85%) copies. The corresponding ALLPATHS assembly has a single copy. The HM056 Alpaca assembly has 26 copies, including one discontinuous copy, on 4 scaffolds. The HM340 Alpaca assembly has 30 copies on 5 scaffolds. To validate this family size disparity relative to the reference, Illumina reads from each accession were mapped to a single copy of the CRP gene from the corresponding assembly. Coverages were compared to upstream and downstream control genes having highly similar GC content and identified as single-copy genes in the Medicago reference genome. In each of the 4 accessions, the Medtr8g031540 sequence had mapping rates that were 11.5 to 26 times greater than expected if this were a single copy gene (Table 4). The extrapolated copy number for each accession is consistently higher than the ALLPATHS but lower than the Alpaca content. Analysis by qPCR also confirmed the multi-copy nature of this CRP, also with the extrapolated copy numbers higher than the ALLPATHS but lower than the Alpaca. Interestingly, both validation methods predicted that the A17 accession has more copies of this gene than annotated in the Mt4.0 reference assembly, which was produced by enhancing an ALLPATHS assembly with additional data [30]. This analysis indicates that each accession has multiple genes from this subfamily though it does not resolve the precise gene copy number per accession. The analysis further indicates that Alpaca overrepresented the gene copy number while the other assemblers underrepresented it in each accession.

**Table 4:** Gene copy number predictions and validations for a CRP3710 subfamily

accession:	HM101	HM034	HM056	HM340
A. Assembly				
MT4.0	2			
ALLPATHS		1	3	3
PBJelly		8	5	3
Alpaca		29	26	30
B. Coverage (RPM)				
Medtr1g061160 (control 1)	0.26	0.38	0.29	0.50
Medtr1g080770 (control 2)	0.29	0.59	0.51	0.57
CRP3710	7.00	5.60	9.00	14.00
estimated copy number	25.5	11.5	22.5	26.2
C. qPCR				
estimated copy number	12.0	11.0	9.7	8.9

A. Annotation found between 1 and 30 copies per assembly. B. Coverages in reads per million bases for this gene and two controls, followed by the copy numbers estimated by fold increase of gene over control average, per accession. C. Copy numbers estimated from quantitative PCR per accession

### Requirements

Alpaca requires ALLPATHS [10], ECTools [33], Flash [37], Bowtie2 [38], and Celera Assembler [16–18]. Its Illumina processing, prior to long-read correction, used 3 K cpu hr. on a 32-core 512 GB RAM resource. The scaffold formation, following long-read correction, used 2 K cpu hr. on a 16-core, 256 GB RAM resource for up to 3 weeks. The long-read correction step used 90 K cpu hr. on up to 100 single-core 2 GB grid resources. The correction step computation is thus costly. It operates on each long read separately so the cpu load should drop linearly with coverage. To evaluate the effect of reducing long-read coverage, the Medicago accessions were partially re-assembled with portions of their corrected long reads. The outcomes were evaluated by unitig size, i.e. the contiguity prior to SimuMate integration, which correlates with the final contig size. Unitig sizes dropped by 14% using three-quarters of the reads and by 49% using half the reads coverage (Additional file 17).

## Discussion

Efficient and accurate de novo assemblies of genomes will greatly facilitate investigation of the functional importance and evolution of copy number variation within and among species [39]. We have presented a new open-source hybrid assembler, Alpaca, that integrates PacBio long-read data with Illumina short-read data to produce high quality contigs and scaffolds. We evaluated Alpaca on one rice genome with 33X long-read coverage, and three *Medicago* genomes using 20X long-read coverage. Alpaca made use of an ALLPATHS-LG [10] assembly and the Illumina short reads required by ALLPATHS. Compared to the short-read assembly that it used as input, Alpaca not only improved the contig contiguity of each genome, it also improved the structural accuracy. This was shown by alignment N50 length and tandem repeat content. Also, Alpaca was able to recover high-copy number variants that were missing from ALLPATHS assemblies and even the *Medicago* reference genome.

As its name was chosen to indicate, Alpaca is a hybrid assembler that relies on the ALLPATHS and Celera Assembler (CA) [16–18] genome assembly pipelines. Alpaca first corrects the high base-call error expected in the PacBio reads. This read-correction step alters the sequences of individual PacBio reads using their alignments to the ALLPATHS short read contigs by running the ECTools [33] software. Alpaca then assembles the corrected long reads into preliminary contigs using Celera Assembler. In parallel, Alpaca relies on ALLPATHS to build scaffolds from the SIPE and LIPE short reads. Alpaca then samples the scaffold sequences to generate synthetic long range mate-pair sequences, and maps those pairs to the long-read contigs. Finally, Alpaca invokes Celera Assembler to generate scaffolds. This strategy relies on long reads early in the pipeline for contig formation. Since long reads form the basis of its contigs, Alpaca is positioned to avoid collapsing tandem repeats already spanned by long reads.

By several measures, the Alpaca assemblies represented improvements over short-read assemblies (by ALLPATHS) and long-read extensions to them (by PBJelly [28]). The Alpaca assemblies were far from perfect, however. In comparison to the high-quality rice reference genome sequence, the Alpaca assembly contained thousands of differences (Table 1). Repeats are the main challenge to genome assembly because they can collapse (i.e. co-assemble into fewer copies) and falsely join sequences on either side of different repeat copies [6]. In our analysis of rice assemblies, we indicated several ways that repeats could be present-but-underrepresented in Alpaca assemblies. We found that 12% of genome repeats were underrepresented in the Alpaca rice assembly, that 29% of assembled repeats were not confirmed in the reference, and that Alpaca captured fewer short repeats than PBJelly (Table 2). Alpaca would not assemble through clusters of repeats where the clusters are not spanned by multiple long reads, and Alpaca could collapse such clusters thereby reducing the short repeat count. The input long-read coverage gets reduced unevenly by the read correction and overlap detection steps, and low-coverage repeats in repeats would induce false joins. Alpaca breaks contigs at positions held together by a single read; a higher threshold would improve accuracy at some cost to contiguity. Finally, the low coverage observed anecdotally at some tandem repeats suggest a cause of phantom repeat instances. Alpaca scaffolds may contain extra repeat instances formed entirely of long reads that (due to coincident sequence errors) align more closely to each other than to the repeat consensus, and this problem would become more prevalent in higher-multiplicity repeats. Higher quality assemblies could probably be obtained, at higher cost, using higher long read coverage, and possibly by also incorporating physical map technology, e.g. [40, 41], but Alpaca provides a lower-coverage option for genome assembly.

We compared Alpaca results to those of the hybrid assembler, PBJelly. Published in 2012, PBJelly set a standard and has been used in more than 200 projects. For example, the ALLPATHS and PBJelly combination was used, with other tools, in recently published assemblies of *Arabidopsis thaliana* [42], *Brassica juncea* [43], and Atlantic cod [44]. The gap-filling approach seems predisposed to perpetuate mis-assemblies, if present, such as the collapse of nearby repeats within a contig. In our comparisons using rice and *Medicago,* Alpaca captured more tandem repeats of sequences 2Kbp and longer. Thus, Alpaca captured more of the gene-length tandem repeats that are difficult to assemble correctly from short reads. PBJelly was much faster as it did not require Alpaca’s CPU-intensive long-read correction step or its Celera Assembler scaffolder step. Alpaca’s substantial computational burden must be weighed against its repeat detection capability and low coverage requirement.

Alpaca is one of several recently-developed hybrid assemblers that incorporate long reads prior to scaffold formation. The hybridSPAdes software for bacterial genomes maps long reads to an assembly graph prior to contig formation [45]. The MaSuRCA software aligns super-reads and mega-reads derived from Illumina and PacBio reads in a form of correction prior to assembly. MaSuRCA assembled a 4 Gbp wheat genome, an order of magnitude larger than rice or Medicago, using 38X PacBio and 110,000 CPU hr. [46]. The non-hybprid assemblers Falcon [25] and Canu [19] use only long reads to generate large, high-quality contigs but their CPU and coverage requirements are high. The DBG2OLC hybrid assembler, which avoids correcting base calls in reads prior to assembly, generated a 2 Mbp contig N50 on *Arabidopsis* using 20X PacBio, though false joins were a concern [47]. The approach was refined for *Drosophila melanogaster* and also merged with other approaches to generate N50 s over 10 Mbp with 50X and above.

Future work remains to refine and accelerate Alpaca. Its algorithms might make use of new methods for locality-sensitive hashing [22] or compressed-read alignments [47] to make it faster. With limited long read coverage available to it, Alpaca is unlikely to provide the completeness and accuracy of assemblies from high-coverage long reads. Indeed, as shown in Table 4, Alpaca can incorporate defects such as low levels of overstated tandem repeat content. It is possible that those defects could be detected and filtered or repaired in software through coverage analysis of reads mapped to the assembly, and this is left for future work.

In this study, four genomes were each assembled using a particular sequencing strategy involving moderate coverage in short reads and low coverage in long reads. This strategy was not necessarily optimal for any of the assemblers tested. With its low requirements for library construction and coverage depth, the strategy applied here could be particularly useful for studies of non-model species genome projects for which limited resources are available. Alpaca appears useful for investigating population variation in tandem repeats and copy number variation in multigene families, thereby enabling studies of expansion and contraction of multi-gene gene families. For studies that rely on de novo assemblies of multiple genomes across a population, the Alpaca strategy permits examination of multiple genomes using moderate levels of long-read sequencing. For projects that already have short-read assemblies of multiple genomes, the Alpaca approach could provide insights into likely shortcomings of those assemblies and assist the selection of specific genomes to target with high-coverage long-read sequencing.

## Conclusions

The Alpaca hybrid genome assembly pipeline uses low-coverage, corrected long reads for contig formation, short reads for consensus accuracy, and long-insert pairs (indirectly) for scaffold formation. On rice and Medicago genomes, Alpaca increased tandem repeat capture relative to two other assembly pipelines. Alpaca appears useful for surveys of copy number variation within multigene families.

## Methods

### Sequence data

The rice (*Oryza sativa* Nipponbare) reference [29, 48] version 4.0, GCA_000005425.2_Build_4.0, was downloaded from NCBI. The rice genome was sequenced by Illumina HiSeq to 50X SIPE with 180 bp inserts and to high coverage of LIPE with each of 2Kbp and 5Kbp inserts as previously described [13]. The LIPE was sampled to 30X per insert size. The genome was further sequenced by PacBio RS II to 34X. The *Medicago truncatula* A17 reference [30, 31] version Mt4.0 was downloaded from JCVI (medicago.jcvi.org/medicago/). Three accessions of *M. truncatula* (HM340, HM056, and HM034) were sequenced by Illumina HiSeq to generated on Illumina HiSeq and PacBio RSII platforms. For rice, Illumina library construction targeted 180 bp SIPE inserts and LIPE inserts at 2 Kbp and 5 Kbp. For Medicago, Illumina library construction targeted 150 bp or 180 bp SIPE and 9Kbp LIPE. All long-read sequencing was performed using mostly P4C2 chemistry with some P5C3 after Blue Pippin size selection. Across accessions, the sequence data provided 48X to 66X SIPE, 51X to 65X LIPE, and 20X to 22X PacBio coverage (Additional file 18).

### Alpaca assembly

**Fig. 2:**
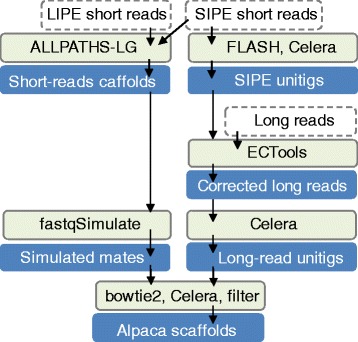
Alpaca pipeline schematic. The figure shows inputs (dashed outline), processes (light-filled boxes), and outputs (blue boxes)

The Alpaca assembly process proceeds through two major parallel assembly phases from deep coverage short sequencing and low coverage long read sequencing, respectively, until finally all data are combined and assembled into scaffold sequences (Fig. 2).

The Illumina SIPE and LIPE data are assembled with ALLPATHS-LG. In parallel, the Illumina SIPE is assembled to unitigs (preliminary contigs). The reads are processed with FLASH v1.2.6 [37] to combine overlapping read pairs into extended, unpaired sequences. The extended sequences output by FLASH in FASTQ format are assembled with Celera Assembler (CA) after converting to the CA FRG format using the fastqToCA utility. FLASH’ed reads were assembled with CA v8.3 with algorithmic parameter settings merSize = 22, merDistinct = 0.99, doOBT = 0, ovlErrorRate = 0.03, doFragmentCorrection = 0, unitigger = bogart, utgGraphErrorRate = 0.02, utgGraphErrorLimit = 2.25, utgMergeErrorRate = 0.03, utgMergeErrorLimit = 4.25, doExtendClearRanges = 0.

Next, the PacBio reads are subject to base call correction using the ECTools correction software. ECTools ran on PacBio filtered sub-reads ≥3000 bp with the Illumina reads pre-assembled by CA. In these experiments, ECTools version e894ba2 was configured, in its correct.sh script, to generate alignments by ‘nucmer --maxmatch -l 15 -b 1000 -g 500’ using nucmer v3.1 within the MUMmer v3.23 package [32]. Other settings used default values. ECTools was run in parallel on partitions of 20 PacBio sequences each and the output was concatenated into a FASTA file of corrected PacBio reads that was saved for the next step. The post-ECTools corrected PacBio reads provided between 7.2 and 9.8X genome coverage for each Medicago accession (9.8X on HM034, 7.2X on HM056, 7.5X on HM340); the rice post-ECTools corrected PacBio reads were filtered to about 10X by using reads of length ≥ 4000 bases. After correction, the corrected PacBio reads are assembled to contigs and scaffolds using CA and the run_alpaca.sh script provided with Alpaca.

Using the output FASTA from the ECTools, the corrected PacBio reads are assembled to unitigs with CA using parameters merSize = 22, ovlMinLen = 500, unitigger = bogart, utgGraphErrorRate = 0.01, and stopAfter = utgcns to stop the assembly process after it computes the consensus for each unitig.

Next, the ALLPATHS scaffold FASTA is used to scaffold the contigs produced from the error corrected PacBio reads. The PacBio reads had so far not been subject to scaffolding since they consist of contiguous and unpaired reads. It is not desirable to directly merge the ALLPATHS and CA assemblies since the assemblies may have significant disagreement. Instead, Alpaca samples synthetic long-range mate-pairs from the ALLPATHS scaffolds so that it can integrate the connectivity information into CA for scaffolding. To do so, Alpaca uses the CA fastqSimulate utility parameterized to generate up to 200X of 2 × 2000 bp pairs with insert sizes 10Kbp, 40Kbp, and 160Kbp. The simulated sequences are mapped to the unitigs using bowtie2 v2.2.3 [38] with parameters ‘--end-to-end --sensitive’. After sampling and mapping, CA is re-started mid-assembly so that it builds contigs and scaffolds from the previously assembled PacBio-only unitigs plus ALLPATHS-derived synthetic-mate pairs, and runs to completion.

Finally, from the CA outputs, scaffold sequences are filtered to remove scaffolds with fewer than 15 reads and to split scaffolds at any position with less than 2X PacBio coverage.

### Alternative assemblies

To evaluate of ALPACA performance, we used ALLPATHS-LG and PBJelly to construct assemblies with the same sequence data sets. The rice assembly was made using ALLPATHS-LG R41348 with MIN_CONTIG = 300. The *M. truncatula* assemblies were made using R49962 (for HM340) or R48288 (HM056 and HM034) with default parameters. The PBJelly assemblies were generated by mapping and layering uncorrected PacBio filtered subreads on ALLPATHS assemblies. PBJelly 14.9.9 was run with the recommended default parameters and configured to call BLASR 1.3.1.140182 [49], reading FASTQ and writing SAM formats, with parameters ‘-minMatch 12 -minPctIdentity 75 -bestn 1 -nCandidates 20 -maxScore −500 -nproc 16 -noSplitSubreads’. For contig size comparisons, contigs were extracted from scaffold files using a uniform rule: from each assembler’s output scaffold FASTA file, sequences were split into contigs at every span of 20 or more consecutive Ns with those Ns removed.

### Reference alignment and accuracy analysis

Raw and corrected reads were aligned to the reference for evaluation using BLASR with parameters “-minReadLength 500 -minMatch 14 -bestn 1 -clipping soft –noSplitSubreads -sam”. Assemblies were evaluated by aligning scaffold sequence to the reference genome of each species using MUMmer’s nucmer local aligner with default parameters [32]. Nucmer alignments were filtered with ‘delta-filter -r’ to produce the ‘df-r’ set. This MUMmer filter chooses the best alignment per reference position determined by the LIS dynamic programming algorithm weighted by the length and identity of the alignments. In *Medicago*, where the reference represents a different accession than those we assembled, nucmer alignments were filtered with ‘delta-filter -q’ to choose the best alignment per assembly position, though comparative results were not affected by this choice. The glocal aligner ATAC [17] version 2008 was run with default parameters. ATAC outputs aggressive and stringent alignments as lines starting with ‘M c’ and ‘M r’ respectively; the aggressive alignments are shown unless otherwise noted. In all cases, alignment lengths were measured in reference coordinates. Quast 4.1 [35] was run with default parameters.

### Tandem repeat identification and analysis

Rice assemblies were evaluated by counting tandem repeats and comparing to the reference. To identify tandem repeats by the “alignment-to-self” method, each assembly (or reference) self-alignment was generated with ‘nucmer –maxmatch –nosimplify’ filtered to retain only alignments of one scaffold (or chromosome) to itself, to retain each A-to-B alignment but exclude its B-to-A mirror, to retain alignments with at least 95% sequence identity, to exclude aligned pairs whose repeat units overlap, and to retain alignments with at most 1Mbp separation at their midpoints. Each assembly was aligned to the reference with ‘nucmer –maxmatch –nosimplify’ and tested for alignments covering 50% of the length of each tandemly repeated unit. The process is automated by the *repeat_content.pl* script in the Alpaca package. Medicago assemblies were characterized by counting tandem repeats as above but without comparison to a reference.

### Identification of tandem gene clusters

Protein-coding sequences were extracted from each ALLPATHS and Alpaca assembly for each *Medicago* accession. All-against-all blastp [50], with parameter “-evalue 1e-5”, was performed on each protein set. Blastp output was processed to replace E-values of 0 with the lowest non-zero E-value in that file, and converted to tabular format. Sequence similarity-based clustering was computed with MCL [51] with command line parameter “-te 4 -I 2.0” for each accession. If two genes in a cluster were spaced by no more than one gene, excluding transposable elements (TEs) present in the reference annotation, a tandem gene pair was called. Tandem gene clusters were obtained by first creating an undirected network using all tandem gene pairs as edges, then extracting all connected-components from the network yielding tandem gene clusters of different sizes. The distribution of different sizes of tandem gene clusters were compared between each ALLPATHS and Alpaca assembly for each Medicago accession.

### Validation of tandem gene family expansion

The tandem copy number expansion of a CRP gene, Medtr8g031540.1, identified in the Alpaca assemblies, was validated by depth of coverage of raw Illumina read mapping counts. A bowtie2 index was created for a homologous 200 bp region, plus 100 bp of flanking sequencing on each end, that fell completely within the CRP gene in the reference as well as the three Alpaca assemblies. For each accession, approximately 15 M Illumina 90–100 bp reads were then mapped via bowtie2 (default parameters) to the 400 bp region. The resulting BAM file was filtered, with samtools view, back to the central 200 bp region in order to obtain all reads that overlapped the region by at least 1 bp. As a control, the same index building and read mapping procedures were applied to 200 bp regions selected within each of two control genes (Medtr1g061160 and Medtr1g080770) that are single copy within Medicago Mt4.0, and each of the three Alpaca assemblies, and in the *Arabidopsis thaliana* genome.

Further validation used qPCR to estimate the relative DNA copy number of the CRP gene compared to the single copy reference genes. All qPCR reactions used the iTAqTM Universal SYBR Green Supermix kit (BioRad, Hercules, CA, USA) and were run on an Applied Biosystems (Foster City, CA, USA) Step One Plus Real-Time PCR thermal cycler with primers designed to amplify a 200 bp product (primer sequences in Additional file 19). Leaf tissue from six plants from each of the four accessions were independently assayed with three technical replicates per plant. Standard curves for each of the four primer sets (two primer sets were used for the CRP gene) were determined by running qPCR on a serial dilution (1×, 2×, 4×, 8×, 16×, and 32×) of pooled template DNA samples, replicated twice. The following thermal cycling conditions were used for all reactions: 95 °C for 20 s, followed by 40 cycles of 95 °C for 3 s and 60 °C for 30 s, followed by melting curve analysis. The CT values were determined using the Applied Biosystems software [52]. Differences in primer efficiency were corrected by multiplying the CT value of each reaction by the slope of the regression across the serial dilutions. Copy number estimates of the CRP for each genotype were determined by 2^[(corrected CT of the reference primer)-(corrected CT of the CRP primer)]. The final copy number in each accession was estimated by averaging the copy number estimates derived from all primer combinations and all six plants per genotype.

## Additional files

Additional file 1: Rice assembly size statistics. (XLSX 42 kb)

Additional file 2: Rice alignment statistics from Nucmer. (XLSX 43 kb)

Additional file 3: Rice alignment statistics from ATAC. (XLSX 41 kb)

Additional file 4: Rice alignment statistics from Quast. (XLSX 36 kb)

Additional file 5: Rice tandem repeat statistics. (XLSX 41 kb)

Additional file 6: Medicago assembly size statistics. (XLSX 37 kb)

Additional file 7: Medicago alignment statistics from Nucmer. (XLSX 54 kb)

Additional file 8: Medicago repeat statistics. (XLSX 48 kb)

Additional file 9: Medicago coverage histogram for mapped raw reads. (PDF 107 kb)

Additional file 10: Medicago gene family size histograms. (PDF 217 kb)

Additional file 11: CRP genes in Medicago assemblies. (PDF 48 kb)

Additional file 12: TE genes in Medicago assemblies. (PDF 39 kb)

Additional file 13: NBS-LRR genes in Medicago assemblies. (PDF 25 kb)

Additional file 14: Phylogeny for CRP0355 in Medicago assemblies. (PDF 442 kb)

Additional file 15: Phylogeny for CRP3710 in Medicago assemblies. (PDF 285 kb)

Additional file 16: Phylogeny for CRP4180 in Medicago assemblies. (PDF 244 kb)

Additional file 17: Coverage titration results. (PDF 22 kb)

Additional file 18: Descriptions of reads used for assemblies. (PDF 79 kb)

Additional file 19: Primer sequences used for CRP amplification. (PDF 39 kb)

Additional file 20: Assembly access instructions. (PDF 55 kb)

## Abbreviations

A17 HM034 HM056 HM340: Accessions for different members of the *Medicago truncatula* species
CPU hr.: One hour of dedicated time on a computer’s central processing unit
CRP: A class of disease-resistance genes encoding cysteine-rich peptides
LIPE: Long-insert paired-end short-read sequencing, also called jumping library or mate pair
N50, NG50: The size-weighted median i.e. the size of smallest span among the minimal set of spans that collectively cover at least half the total span (N50) or half the assumed genome size (NG50)
NBS-LRR: A class of disease-resistance genes encoding nucleotide-binding site leucine-rich repeat proteins
qPCR: Quantitative polymerase chain reaction
SIPE: Short-insert paired-end short-read sequencing
TE: Transposal element
