# Supplementary material for: Hybrid assembly with long and short reads improves discovery of gene family expansions
Source: BMC Genomics. 2017 Jul 19;18:541. doi: 10.1186/s12864-017-3927-8 (PMC5518131; doi:10.1186/s12864-017-3927-8)
Supplement: Supplementary file 9 — Medicago coverage histogram for mapped raw reads. (PDF 107 kb) [file 12864_2017_3927_MOESM9_ESM.pdf]

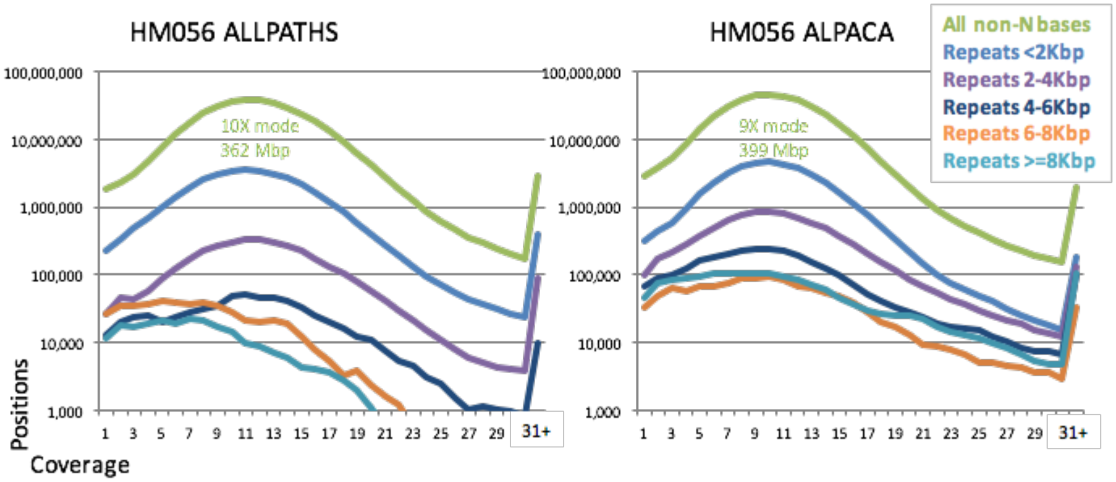

Supplemental Figure S1. Coverage distributions for same-scaffold repeats. Uncorrected long reads were mapped to ALLPATHS and Alpaca assemblies of *M. truncatula* HM056. Horizontal axis: bins representing scaffold positions covered by 1 or more reads. Vertical axis: Number of assembly positions having the given coverage. Colors: coverage over the genome as a whole (green) or over repeats binned by size.
