# Supplementary material for: Hybrid assembly with long and short reads improves discovery of gene family expansions
Source: BMC Genomics. 2017 Jul 19;18:541. doi: 10.1186/s12864-017-3927-8 (PMC5518131; doi:10.1186/s12864-017-3927-8)
Supplement: Supplementary file 10 — Medicago gene family size histograms. (PDF 217 kb) [file 12864_2017_3927_MOESM10_ESM.pdf]

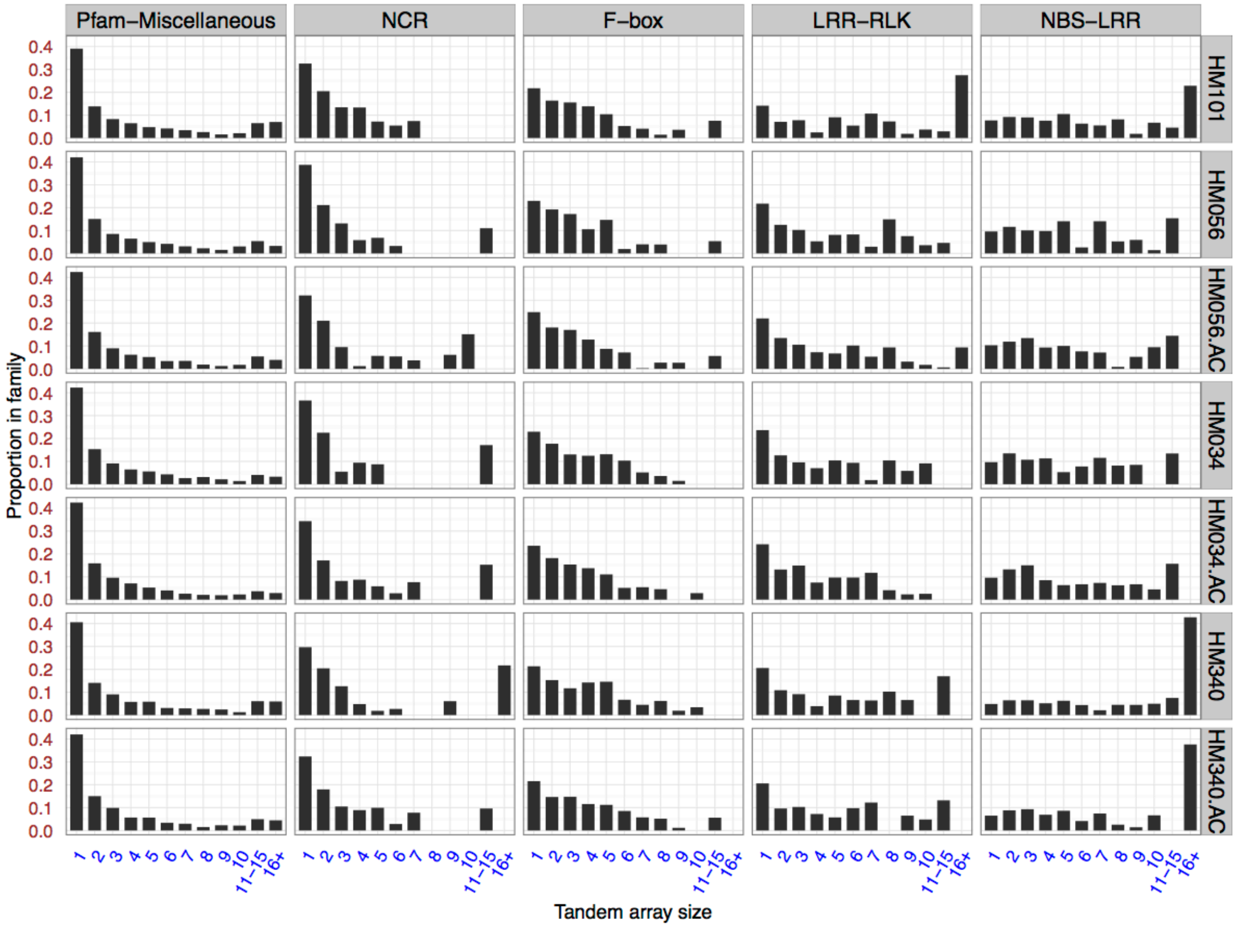

Supplemental Figure S2. Seven assemblies of four *Medicago truncatula* accessions were analyzed for gene cluster content related to four gene families: NCR, F-box, LRR-RLK, NBS-LRR. A miscellaneous grouping was included for comparison. Each vertical bar of each histogram indicates the portion of tandem gene clusters within a given gene family that contain X genes, where X is displayed on the horizontal axis. HM101 = the Mt4.0 reference assembly of the A17 (HM101) accession, HM056 = the ALLPATHS assembly of the HM056 accession, HM056.AC = the Alpaca assembly of the HM056 accession, etc. The “AC” suffix in an assembly name indicates the assembly was generated with Alpaca.
