# Supplementary material for: Hybrid assembly with long and short reads improves discovery of gene family expansions
Source: BMC Genomics. 2017 Jul 19;18:541. doi: 10.1186/s12864-017-3927-8 (PMC5518131; doi:10.1186/s12864-017-3927-8)
Supplement: Supplementary file 11 — CRP genes in Medicago assemblies. (PDF 48 kb) [file 12864_2017_3927_MOESM11_ESM.pdf]

**Family: CRP**	red for drop more than 1				green for diff >= 100%			
	yellow for increase more than 1				blue for diff <= -100%			
	**Mt4.0**	**ALLPATHS**	**ALPACA**	**ALLPATHS**	**ALPACA**	**ALLPATHS**	**ALPACA**	**Average**
**sub-family**	**HM101**	**HM034**	**HM034**	**HM056**	**HM056**	**HM340**	**HM340**	**Difference**
CRP0000	9	8	8	9	8	9	9	-0.33
CRP0010	11	9	11	14	13	12	11	0.00
CRP0030	2	2	2	2	2	2	1	-0.33
CRP0065	2	1	1	2	2	2	2	0.00
CRP0080	4	5	5	5	6	7	7	0.33
CRP0090	1	1	1	0	0	1	1	0.00
CRP0110	28	27	27	24	23	25	26	0.00
CRP0240	0	0	0	0	0	2	1	-0.33
CRP0300	5	7	6	7	7	7	8	0.00
CRP0305	0	0	0	0	0	1	1	0.00
CRP0310	17	13	13	14	14	15	16	0.33
CRP0330	25	23	25	23	24	25	23	0.33
CRP0340	0	1	1	1	1	2	2	0.00
CRP0350	2	3	2	3	2	1	1	-0.67
CRP0355	38	36	54	37	48	35	38	10.67
CRP0360	1	1	1	1	1	1	1	0.00
CRP0500	1	1	1	1	0	1	1	-0.33
CRP0550	2	2	2	3	3	2	2	0.00
CRP0570	1	2	2	1	2	1	1	0.33
CRP0580	2	3	3	3	3	2	2	0.00
CRP0590	1	1	1	1	1	1	1	0.00
CRP0620	1	1	1	0	0	0	0	0.00
CRP0630	1	1	0	1	0	1	1	-0.67
CRP0670	4	4	4	4	4	4	4	0.00
CRP0675	25	23	26	24	26	22	20	1.00
CRP0680	1	1	1	1	1	0	0	0.00
CRP0750	1	1	1	0	0	1	1	0.00
CRP0810	0	0	1	0	0	0	0	0.33
CRP0830	1	1	1	1	1	1	1	0.00
CRP0860	13	14	14	14	12	13	14	-0.33
CRP0940	7	3	3	4	5	5	5	0.33
CRP0980	1	1	1	1	0	2	2	-0.33
CRP1040	1	0	0	1	1	1	0	-0.33
CRP1100	2	2	2	2	2	3	3	0.00
CRP1120	10	8	8	9	9	11	8	-1.00
CRP1130	9	9	7	15	15	5	7	0.00

CRP1140	2	3	4	5	5	6	4	-0.33
CRP1150	5	5	5	9	8	6	6	-0.33
CRP1160	36	21	20	17	21	18	22	2.33
CRP1170	19	16	13	17	17	16	17	-0.67
CRP1180	6	7	6	7	6	5	5	-0.67
CRP1190	96	78	82	84	79	65	63	-1.00
CRP1200	16	15	17	11	10	14	14	0.33
CRP1210	20	23	26	24	26	30	31	2.00
CRP1220	9	7	6	6	5	5	4	-1.00
CRP1230	31	24	30	31	31	26	26	2.00
CRP1240	16	17	22	17	14	20	21	1.00
CRP1250	18	14	13	12	12	14	16	0.33
CRP1260	6	7	7	8	9	9	7	-0.33
CRP1270	5	4	4	5	7	7	7	0.67
CRP1280	2	1	1	2	2	1	1	0.00
CRP1290	8	6	6	4	4	6	7	0.33
CRP1300	16	13	13	14	16	10	9	0.33
CRP1310	3	7	6	6	5	6	8	0.00
CRP1320	5	3	3	6	7	4	5	0.67
CRP1400	11	17	20	14	18	18	13	0.67
CRP1410	15	17	18	14	15	16	18	1.33
CRP1420	19	16	17	14	14	21	19	-0.33
CRP1430	80	72	67	75	75	75	81	0.33
CRP1440	41	40	38	35	34	35	32	-2.00
CRP1450	24	19	19	19	21	16	14	0.00
CRP1460	9	7	10	6	6	8	9	1.33
CRP1470	19	19	21	18	20	22	19	0.33
CRP1480	4	4	3	2	3	5	3	-0.67
CRP1490	5	3	2	3	4	2	2	0.00
CRP1500	10	9	11	10	9	8	8	0.33
CRP1510	8	12	9	11	11	10	11	-0.67
CRP1520	85	82	104	81	111	90	99	20.33
CRP1530	41	23	22	23	20	26	29	-0.33
CRP1600	21	17	18	22	23	16	17	1.00
CRP1605	1	1	1	1	1	1	1	0.00
CRP1640	18	16	17	20	17	19	22	0.33
CRP1650	17	12	14	9	13	15	16	2.33
CRP1660	2	1	1	1	1	2	1	-0.33
CRP1700	16	10	10	9	11	10	9	0.33
CRP1710	1	3	2	3	2	1	1	-0.67

CRP1730	18	20	17	18	18	17	17	-1.00
CRP1740	4	3	2	3	3	2	2	-0.33
CRP1760	5	5	5	5	5	5	5	0.00
CRP1810	3	3	5	4	5	2	3	1.33
CRP1820	1	0	0	1	0	0	0	-0.33
CRP1830	0	1	1	0	0	1	1	0.00
CRP1850	1	2	2	2	2	1	1	0.00
CRP1860	1	1	1	2	2	0	0	0.00
CRP1870	1	1	1	1	1	1	1	0.00
CRP1885	0	0	0	0	0	1	1	0.00
CRP1910	1	1	1	1	1	1	1	0.00
CRP1920	1	1	1	1	1	1	1	0.00
CRP1930	4	4	4	3	3	4	4	0.00
CRP1950	0	0	0	0	0	1	0	-0.33
CRP1990	2	1	1	1	2	1	1	0.33
CRP2040	1	1	1	1	1	1	1	0.00
CRP2310	2	3	3	3	3	3	3	0.00
CRP2350	16	7	15	9	14	6	10	5.67
CRP2360	6	1	4	3	5	4	4	1.67
CRP2370	1	1	1	1	0	0	0	-0.33
CRP2420	1	1	1	1	1	1	1	0.00
CRP2440	1	0	0	0	0	0	0	0.00
CRP2480	2	1	1	2	2	5	5	0.00
CRP2490	1	1	1	1	1	1	1	0.00
CRP2510	2	1	1	2	1	1	1	-0.33
CRP2700	11	16	14	15	12	14	14	-1.67
CRP2820	1	1	1	1	1	1	1	0.00
CRP2840	3	1	1	1	2	3	3	0.33
CRP2850	5	5	5	5	5	5	6	0.33
CRP2855	6	7	7	7	8	9	9	0.33
CRP2860	3	5	5	6	6	3	4	0.33
CRP2960	1	1	1	1	1	1	1	0.00
CRP2980	2	2	2	2	1	2	2	-0.33
CRP3020	4	3	3	3	3	4	3	-0.33
CRP3080	15	11	16	11	12	11	15	3.33
CRP3150	15	15	15	15	15	12	12	0.00
CRP3210	3	2	1	3	3	3	3	-0.33
CRP3330	2	2	2	2	2	2	1	-0.33
CRP3350	2	2	2	2	2	2	2	0.00
CRP3360	11	7	10	9	9	12	11	0.67

CRP3370	11	8	15	8	15	7	17	8.00
CRP3375	1	1	1	1	1	1	1	0.00
CRP3380	4	3	4	4	4	3	4	0.67
CRP3390	1	1	1	0	1	0	0	0.33
CRP3410	2	3	3	3	3	2	2	0.00
CRP3420	1	1	1	1	1	1	1	0.00
CRP3430	1	1	1	1	1	1	1	0.00
CRP3440	1	1	2	2	2	1	3	1.00
CRP3460	2	1	1	2	2	1	1	0.00
CRP3480	10	9	9	8	9	8	8	0.33
CRP3495	1	1	1	1	1	1	1	0.00
CRP3500	3	2	3	3	3	3	4	0.67
CRP3510	1	2	1	2	1	1	1	-0.67
CRP3610	4	5	4	5	5	5	5	-0.33
CRP3650	6	2	3	3	4	4	4	0.67
CRP3710	7	8	34	8	30	6	32	24.67
CRP3730	1	0	0	0	0	0	0	0.00
CRP3800	1	1	1	1	1	1	1	0.00
CRP3860	7	7	7	7	8	6	7	0.67
CRP3865	19	20	21	21	19	22	22	-0.33
CRP3870	1	1	1	1	1	1	1	0.00
CRP3980	3	3	3	4	4	3	3	0.00
CRP3990	1	1	1	2	1	1	1	-0.33
CRP4050	1	1	1	1	1	1	1	0.00
CRP4060	1	1	1	1	1	1	1	0.00
CRP4140	2	2	2	2	2	2	2	0.00
CRP4180	11	6	19	7	13	7	26	12.67
CRP4210	2	2	2	2	2	2	2	0.00
CRP4220	5	4	4	5	5	5	5	0.00
CRP4240	6	6	6	6	7	6	6	0.33
CRP4260	0	1	1	0	0	0	1	0.33
CRP4380	1	1	1	1	1	1	1	0.00
CRP4410	1	1	1	1	1	1	1	0.00
CRP4430	2	1	1	1	1	1	1	0.00
CRP4540	1	1	1	1	1	1	1	0.00
CRP4580	3	3	3	3	3	3	3	0.00
CRP4610	1	1	1	1	0	0	1	0.00
CRP4625	6	2	3	5	7	3	3	1.00
CRP4630	9	9	10	9	9	9	8	0.00
CRP4640	1	1	1	1	1	1	1	0.00

CRP4660	5	4	3	5	4	4	4	-0.67
CRP4670	4	4	5	4	5	5	5	0.67
CRP4680	2	2	2	2	2	2	2	0.00
CRP4690	1	1	2	1	2	4	3	0.33
CRP4730	2	2	2	2	2	2	2	0.00
CRP4750	2	2	2	2	2	2	2	0.00
CRP4780	0	0	0	0	0	1	1	0.00
CRP4800	0	0	0	0	0	0	1	0.33
CRP4810	10	12	11	10	8	9	9	-1.00
CRP4820	11	10	13	8	9	8	9	1.67
CRP4920	5	6	6	6	6	7	7	0.00
CRP5010	3	2	3	3	2	2	2	0.00
CRP5310	2	2	2	2	2	2	2	0.00
CRP5500	2	0	0	1	1	1	1	0.00
CRP5545	1	2	2	1	1	2	2	0.00
CRP5600	2	2	2	2	2	3	3	0.00
CRP5650	1	1	1	1	1	1	1	0.00
CRP5660	3	3	3	3	3	3	3	0.00
CRP5680	3	4	3	5	5	4	4	-0.33
CRP5730	1	1	1	1	1	1	1	0.00
CRP5800	9	7	7	8	8	8	10	0.67
CRP5940	2	2	2	2	2	2	2	0.00
CRP6020	9	8	9	8	9	7	7	0.67
CRP6120	22	19	29	19	20	18	19	4.00
CRP6130	3	3	3	3	2	2	2	-0.33
CRP6150	13	10	15	12	10	11	15	2.33
CRP6250	1	1	1	1	1	1	1	0.00
