# Supplementary material for: Hybrid assembly with long and short reads improves discovery of gene family expansions
Source: BMC Genomics. 2017 Jul 19;18:541. doi: 10.1186/s12864-017-3927-8 (PMC5518131; doi:10.1186/s12864-017-3927-8)
Supplement: Supplementary file 12 — TE genes in Medicago assemblies. (PDF 39 kb) [file 12864_2017_3927_MOESM12_ESM.pdf]

Family: TE

		red for drop more than 1<br>yellow for increase more than 1				green for diff >= 100%<br>blue for diff <= -100%		
**sub-family**	**Mt4.0 HM101**	**ALLPATHS HM034**	**ALPACA HM034**	**ALLPATHS HM056**	**ALPACA HM056**	**ALLPATHS HM340**	**ALPACA HM340**	**Average Difference**
RNase_H	39	51	58	43	47	42	37	2.00
RVP	1	1	1	0	0	1	2	0.33
RVT_1	1791	1494	2495	1588	2350	1465	1918	738.67
zf-CCHC	94	123	148	139	173	111	113	20.33
VPR	1	1	1	1	1	0	0	0.00
Vpu	0	0	0	1	0	0	0	-0.33
Phage_integrase	4	0	0	0	0	0	0	0.00
Gag_p24	3	1	1	3	3	5	2	-1.00
rve	660	636	1429	650	1276	618	870	557.00
dUTPase	1	1	1	1	1	1	1	0.00
Transposase_mut	0	0	0	0	0	0	1	0.33
Hepatitis_core	0	0	0	1	0	0	0	-0.33
HTH_Tnp_Tc3_2	5	13	14	6	8	2	1	0.67
DDE_Tnp_Tn3	0	1	1	0	1	1	1	0.33
HTH_Tnp_1	3	0	0	0	0	0	0	0.00
DDE_Tnp_1	1	0	0	0	0	0	0	0.00
zf-BED	26	35	31	31	40	35	33	1.00
Transposase_21	94	81	144	81	116	73	125	50.00
Transposase_22	0	1	1	0	0	1	1	0.00
Transposase_24	97	68	106	70	108	77	110	36.33
Transposase_23	1	0	0	0	1	0	2	1.00
DDE_Tnp_IS66	0	0	0	1	1	0	0	0.00
ATHILA	133	111	338	109	257	132	221	154.67
DBD_Tnp_Mut	95	100	106	103	105	76	85	5.67
DDE_1	33	6	22	7	30	13	27	17.67
HTH_Tnp_Tc5	5	4	5	4	5	5	8	1.67
Gag_spuma	0	1	0	0	0	0	0	-0.33
Retrotrans_gag	662	590	1464	636	1208	621	877	567.33
Transposase_28	6	9	6	7	8	7	8	-0.33
CENP-B_N	0	0	0	1	0	0	0	-0.33
Reo_sigmaC	1	1	4	1	1	3	1	0.33
Plant_tran	107	75	112	76	109	75	100	31.67
DUF659	31	31	34	32	32	23	29	3.00
DUF746	0	1	1	0	0	0	0	0.00
Dimer_Tnp_hAT	153	132	159	140	163	106	112	18.67
Zea_mays_MuDR	1	1	1	1	0	1	1	-0.33
RVT_thumb	0	0	0	0	1	0	0	0.33
BCNT	1	1	1	1	1	1	1	0.00
RVT_2	1612	1063	1940	1079	1826	950	1525	733.00

RVP_2	238	238	503	271	462	283	372	181.67
zf-RVT	1658	1395	1453	1467	1445	1210	1219	15.00
RVT_3	1602	1227	1671	1283	1583	1124	1198	272.67
PMD	827	540	961	575	875	524	603	266.67
UBN2	488	490	908	465	835	420	613	327.00
UBN2_2	278	235	346	248	348	227	308	97.33
UBN2_3	205	204	231	213	244	207	236	29.00
PIF1	362	302	360	299	337	266	272	34.00
Herpes_Helicase	85	56	64	69	73	40	39	3.67
MULE	520	359	509	390	520	351	460	129.67
UvrD_C_2	0	1	1	2	1	1	0	-0.67
gag-asp_proteas	111	124	230	143	206	167	173	58.33
gag_pre-integrs	83	85	111	74	103	56	64	21.00
Transpos_assoc	36	34	51	39	53	26	30	11.67
DUF4413	45	42	53	37	44	33	33	6.00
DDE_3	30	14	21	19	27	13	14	5.33
DUF4218	14	24	32	24	37	30	55	15.33
CAF1	12	15	14	15	14	15	15	-0.67
AT_hook	4	7	7	4	3	4	5	0.00
Cauli_VI	3	6	10	5	11	5	6	3.67
zf-CCHC_2	3	4	6	2	2	3	4	1.00
zf-H2C2_2	3	2	2	4	3	2	2	-0.33
DUF4371	97	73	89	71	85	49	54	11.67
DUF4216	11	11	16	19	21	14	15	2.67
zf-C2H2_4	9	7	7	8	8	8	9	0.33
Helitron_like_N	32	31	40	34	40	37	32	3.33
DNA_pol_A_exo1	23	21	21	19	21	21	22	1.00
Intron_maturas2	5	3	5	4	4	3	3	0.67
DUF4219	46	68	89	50	69	46	61	18.33
XRN_N	6	6	6	6	6	6	7	0.33
DUF4283	321	368	391	372	375	318	302	3.33
zf-C2H2_jaz	25	26	27	22	20	25	28	0.67
Asp_protease	3	3	3	1	1	2	2	0.00
Asp_protease_2	26	46	139	35	103	38	45	56.00
DDE_Tnp_4	133	84	108	87	114	78	96	23.00
SWIM	74	65	86	77	99	50	71	21.33
zf-CCHC_4	128	122	120	139	130	101	111	-0.33
Chromo	52	59	89	60	69	44	58	17.67
zf-GRF	117	130	150	124	136	114	133	17.00
Exo_endo_phos	87	90	105	99	98	94	85	1.67
Exo_endo_phos_2	38	35	43	31	28	23	29	3.67
