# Supplementary material for: Hybrid assembly with long and short reads improves discovery of gene family expansions
Source: BMC Genomics. 2017 Jul 19;18:541. doi: 10.1186/s12864-017-3927-8 (PMC5518131; doi:10.1186/s12864-017-3927-8)
Supplement: Supplementary file 13 — NBS-LRR genes in Medicago assemblies. (PDF 25 kb) [file 12864_2017_3927_MOESM13_ESM.pdf]

Family: NBS-LRR

red for drop more than 1 | green for diff >= 100%
yellow for increase more than 1 | blue for diff <= -100%

sub-family	Mt4.0 HM101	ALLPATHS HM034	ALPACA HM034	ALLPATHS HM056	ALPACA HM056	ALLPATHS HM340	ALPACA HM340	Average Difference
cnl0100	8	8	10	8	6	9	15	2.00
cnl0200	38	37	39	34	36	60	60	1.33
cnl0300	9	9	9	10	10	8	8	0.00
cnl0400	46	43	45	38	42	36	37	2.33
cnl0450	24	28	32	29	31	23	26	3.00
cnl0500	4	3	3	3	3	2	3	0.33
cnl0600	15	13	18	11	14	9	7	2.00
cnl0700	24	22	21	22	20	22	22	-1.00
cnl0800	20	18	17	20	20	17	16	-0.67
cnl0850	6	5	5	7	7	8	8	0.00
cnl0900	25	26	26	24	23	24	26	0.33
cnl0950	25	25	28	27	33	32	34	3.67
cnl0960	15	13	13	14	14	12	12	0.00
cnl1000	58	48	47	47	47	42	41	-0.67
cnl1080	5	4	5	7	6	6	6	0.00
cnl1100	8	9	9	8	7	10	9	-0.67
cnl1190	6	15	16	11	11	18	19	0.67
cnl1200	5	6	6	5	5	5	5	0.00
cnl1300	7	6	6	6	6	5	6	0.33
cnl1400	46	38	36	43	46	39	37	-0.33
cnl1500	64	42	42	47	53	38	38	2.00
cnl1600	22	18	20	18	19	16	18	1.67
tnl0100	42	42	40	45	42	46	40	-3.67
tnl0200	12	11	12	11	11	6	5	0.00
tnl0300	6	13	14	7	5	4	4	-0.33
tnl0400	80	57	63	71	75	57	62	5.00
tnl0480	71	64	67	75	75	57	63	3.00
tnl0500	21	27	31	25	24	23	30	3.33
tnl0550	20	14	14	20	22	19	20	1.00
tnl0590	4	4	4	4	4	4	4	0.00
tnl0600	12	10	11	11	12	14	15	1.00
tnl0700	5	5	7	5	6	3	6	2.00
tnl0750	11	7	8	12	10	12	12	-0.33
tnl0800	20	20	22	18	21	19	20	2.00
tnl0850	62	50	54	58	62	74	72	2.00
