# Supplementary material for: Hybrid assembly with long and short reads improves discovery of gene family expansions
Source: BMC Genomics. 2017 Jul 19;18:541. doi: 10.1186/s12864-017-3927-8 (PMC5518131; doi:10.1186/s12864-017-3927-8)
Supplement: Supplementary file 17 — Coverage titration results. (PDF 22 kb) [file 12864_2017_3927_MOESM17_ESM.pdf]

Supplemental table S7. Running Alpaca with lower coverage reduces contiguity.

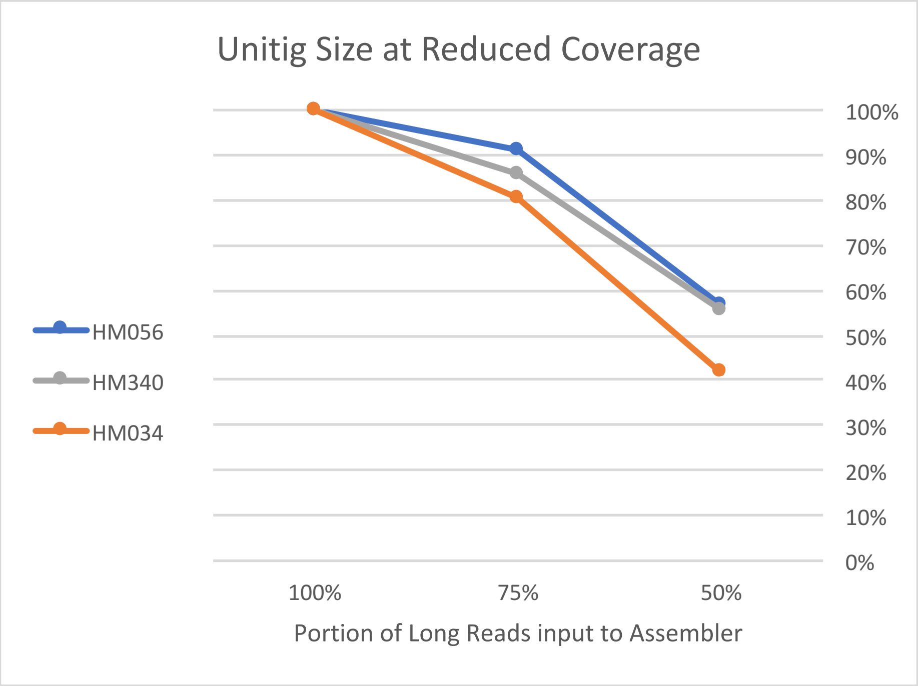

Accession	Contig NG50		100% of reads	75% of reads	50% of reads
HM056	94,385	Mbp input	3,590	2,692	1,795
		Unitig N50	60,169	54,801	34,070
		NG50 pct	100.00%	91.08%	56.62%
HM034	282,425	Mbp input	4,869	3,650	2,438
		Unitig NG50	193,991	155,675	80,792
		NG50 pct	100.00%	80.25%	41.65%
HM340	123,113	Mbp input	3,728	2,797	1,864
		Unitig NG50	75,942	64,996	42,231
		NG50 pct	100.00%	85.59%	55.61%
	**Average**	**NG50 pct**		**85.64%**	**51.29%**

Three Medicago accessions are shown with contig NG50 from the full Alpaca assembly.
At 100% of reads, the total bases input and unitig NG50 output are shown.
Re-run with 75% of reads, the unitig NG50 dropped to 86% its initial value, on average.
Re-run with 50% of reads, the unitig NG50 dropped to 51% its initial value, on average
Every 4th or 2nd read was deleted with the Celera Assembler gatekeeper --edit.
Re-runs were started with Celera Assembler runCA --stopafter=utgcns.
Unitigs were dumped with Celera Assembler tigStore -t tigstore 2 -U -d consensus.
NG50 was computed against the 412,800,391 bp reference genome size.
