# Supplementary material for: Hybrid assembly with long and short reads improves discovery of gene family expansions
Source: BMC Genomics. 2017 Jul 19;18:541. doi: 10.1186/s12864-017-3927-8 (PMC5518131; doi:10.1186/s12864-017-3927-8)
Supplement: Supplementary file 18 — Descriptions of reads used for assemblies. (PDF 79 kb) [file 12864_2017_3927_MOESM18_ESM.pdf]

Accession	Platform	Details	Insert (bp)	Reads (M)	Gbp	Cov	SRA
HM340	Illumina	TruSeq v3	150	166.118	33.2	66X	SRX661000
HM340	Illumina	Nextera Mate Pair	9000	143.968	28.8	58X	SRX682015
HM340	PacBio	P4C2,P5C3	not paired	2.341	10.4	21X	SRX1651452
HM056	Illumina	KAPA HTP	180	121.411	24.3	49X	SRX663485
HM056	Illumina	Nextera Mate Pair	9000	127.976	25.6	51X	SRX675897
HM056	PacBio	P4C2	not paired	1.857	10.7	20X	SRX1672008
HM034	Illumina	KAPA HTP	180	135.693	27.1	54X	SRX679311
HM034	Illumina	Nextera Mate Pair	9000	163.152	32.6	65X	SRX680441
HM034	PacBio	P4C2	not paired	1.565	9.9	20X	SRX1671504
Rice	Illumina	HiSeq 2000 2x101	180	490.000	49.5	101	SRX734432
Rice	Illumina	HiSeq 2000 2x50	2000	4995.000	200.0	408	SRX179260
Rice	Illumina	HiSeq 2000 2x50	5000	3429.000	171.5	351	SRX179265
Rice	PacBio	C1, C2, C3	not paired	4.570	12.8	34X	SRR3743363

Supplemental Table S4. Sequencing data available for assembly. Three accessions of *Medicago truncatula* were subjected to short-read and long-read sequencing at NCGR (www.ncgr.org). Illumina short-insert libraries were prepared with either TruSeq or KAPA kits to target 150bp or 180bp insert sizes, respectively. Illumina long-insert libraries were prepared with Nextera kits to target 9Kbp insert sizes. Short-read sequencing used the HiSeq 2000 platform to produce 2x100bp paired reads. Unpaired long reads were generated on a PacBio RS II instrument with either P4C2 or P5C3 chemistry after size-selection with a Blue Pippin instrument. Sequencing outputs are quantified here by millions of reads and billions of bases delivered by instrument software. Genome coverage estimates the fold-coverage assuming a 500 Mbp genome size. Sequence identifiers are shown for the NCBI Sequence Read Archive (SRA), where all the sequences belong to BioProject PRJNA256006. (Of two HM340 runs with the SRA identifier SRX1651452, the Alpaca assembly used only the SRR3316714 run that provided 21X coverage.) The ALLPATHS-LG assemblies used the short-read sequencing exclusively, while the Alpaca and PBJelly assemblies also used the long reads. The rice Illumina data, and the ALLPATHS assembly of it, have been described previously [Schatz, Maron, et al (2014) DOI:10.1186/PREACCEPT-2784872521277375]. The rice PacBio data was generated at CSHL (www.cshl.org) over several years and several upgrades to the Pacific Biosciences RS II sequencer.
