# Supplementary material for: Hybrid assembly with long and short reads improves discovery of gene family expansions
Source: BMC Genomics. 2017 Jul 19;18:541. doi: 10.1186/s12864-017-3927-8 (PMC5518131; doi:10.1186/s12864-017-3927-8)
Supplement: Supplementary file 19 — Primer sequences used for CRP amplification. (PDF 39 kb) [file 12864_2017_3927_MOESM19_ESM.pdf]

Control 1:
Forward TCATGGAACCAAAACGAGTG
Reverse TCTGTGCGAAAAACTCCAAAG
Control 2:
Forward TTACATGGGAGGACCAGCAG
Reverse CGACAACCTTTGAATACGATTG
Test 1:
Forward ATGATCGGTTTTACTTTGCTTTTC
Reverse TGATTTCCAACAAATACACCAGAG
Test 2:
Forward GGTTTTACTTTGCTTTTCACAACC
Reverse TCTGATTTCCAACAAATACACCAG

Supplemental Table S5. Primers used to amplify the CRP gene cluster.
