# Supplementary material for: Hybrid assembly with long and short reads improves discovery of gene family expansions
Source: BMC Genomics. 2017 Jul 19;18:541. doi: 10.1186/s12864-017-3927-8 (PMC5518131; doi:10.1186/s12864-017-3927-8)
Supplement: Supplementary file 20 — Assembly access instructions. (PDF 55 kb) [file 12864_2017_3927_MOESM20_ESM.pdf]

Genome		COGE ID	URL
Oryza sativa	Nipponbare	30550	https://genomevolution.org/coge/GenomeInfo.pl?gid=30550
M. truncatula	HM034	30539	https://genomevolution.org/coge/GenomeInfo.pl?gid=30539
M. truncatula	HM056	30543	https://genomevolution.org/coge/GenomeInfo.pl?gid=30543
M. truncatula	HM340	30547	https://genomevolution.org/coge/GenomeInfo.pl?gid=30547

Supplemental Table S6. Four Alpaca assemblies are available as scaffold FASTA files from GOGE (https://genomevolution.org) under the COGE identifiers shown.
age difference between the Alpaca gene count and the ALLPATHS gene count.
