# Supplementary material for: Hybrid assembly with long and short reads improves discovery of gene family expansions
Source: BMC Genomics. 2017 Jul 19;18:541. doi: 10.1186/s12864-017-3927-8 (PMC5518131; doi:10.1186/s12864-017-3927-8)
Supplement: Supplementary file 16 — Phylogeny for CRP4180 in Medicago assemblies. (PDF 244 kb) [file 12864_2017_3927_MOESM16_ESM.pdf]

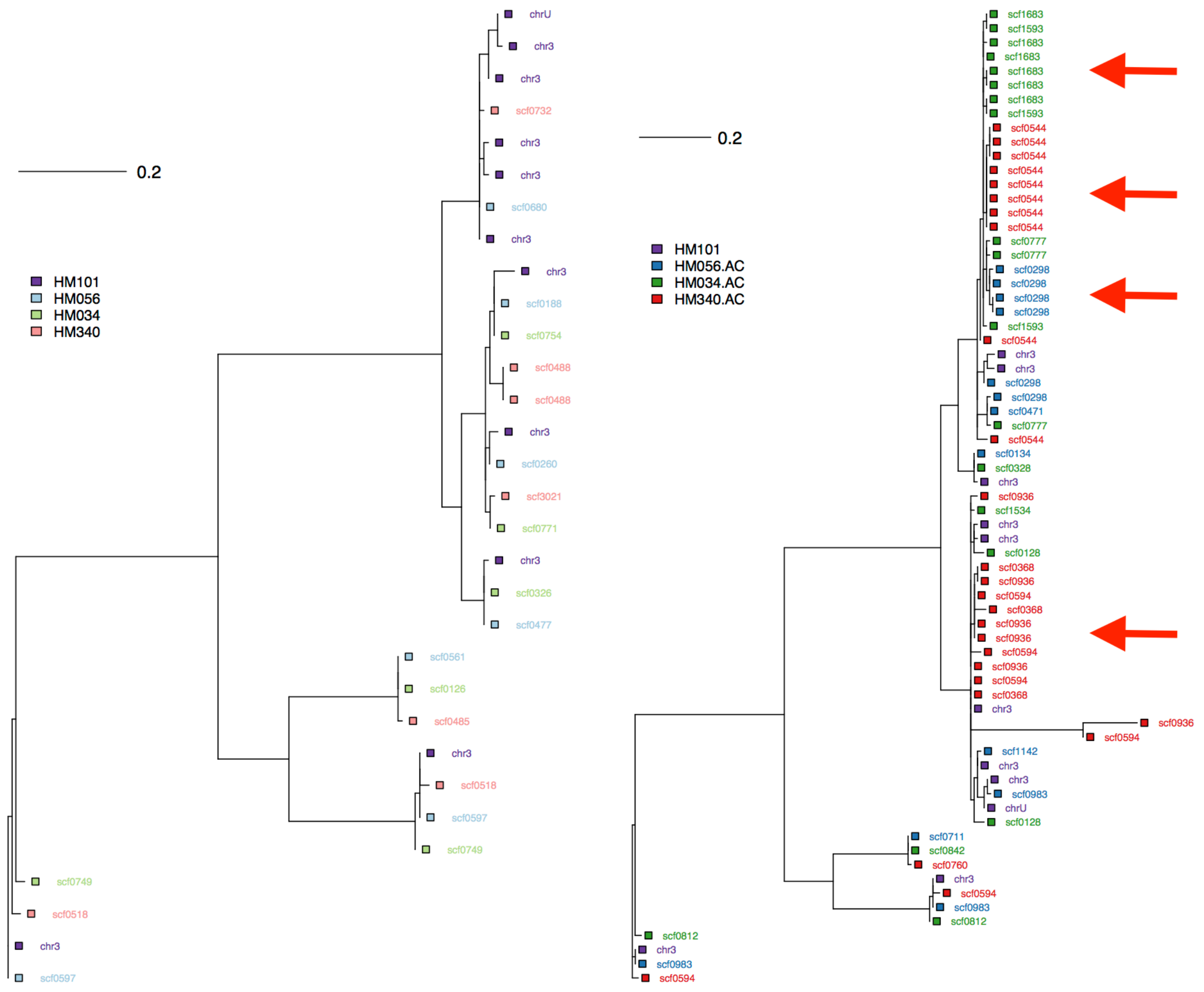

Supplemental Figure S5. Phylogeny reconstruction of the CRP4180 subfamily of the CRP gene family across four accessions of Medicago truncatula based on Mt4.0 reference assembly and three ALLPATHS assemblies (left) or three Alpaca assemblies (right). The Alpaca assembly contains accession-specific gene family expansions absent from the ALLPATHS assembly. Red arrows highlight the largest accession specific expansions.
